# Concentrations, Distributions, and Risk Assessment of HBCD in Sediment in the Weihe River Basin in Northwest China

**DOI:** 10.3390/ijerph15112340

**Published:** 2018-10-23

**Authors:** Xueli Wang, Xiaoyu Yuan, Shengke Yang, Yaqian Zhao

**Affiliations:** 1 Key Laboratory of Subsurface Hydrology and Ecological Effects in Arid Region, Ministry of Education, Chang’an University, Xi’an 710054, China; 15129037687@163.com (X.Y.); ysk110@126.com (S.Y.); 2 Dooge Centre for Water Resource Research, School of Civil Engineering, University College Dublin, Belfield, 999014 Dublin 4, Ireland; yaqian.zhao@ucd.ie

**Keywords:** HBCD, sediment, distributions, risk assessment

## Abstract

As one of the most widely used brominated flame retardants, hexabromocyclododecane (HBCD) is found widely in the environmental media. In this study, the content and spatial distribution of HBCD and risk posed by HBCD in surface sediment in the Weihe River Basin in Northwest China were investigated. The HBCD concentration ranged nd–4.04 ng/g dw with the mean was 0.45 ng/g dw. The major source of HBCD in surface sediment was local point discharge. The distribution profiles of α-, β-, γ-HBCD were 24.7–87.9%, 0–42.0%, and 0–67.1%, respectively. Specially, α-HBCD was the dominating isomer in most sample sites. This differed significantly from that in HBCD technical product, which might be attributed to the different degradation rates and inter-transformation of the three HBCD isomers. Risk quotient method was used to assess the potential risk posed by HBCD in sediment. HBCD do not pose strong risks to aquatic algae organisms in the Weihe River Basin.

## 1. Introduction

Hexabromocyclododecanes (HBCD) is a widely used brominated flame retardants (BFRs) [1,2,3]. Over the past few decades, global production and environmental concentration of HBCD have increased, especially for the strict regulations and banning of some polybrominated diphenyl ethers (PBDE) formulations [4,5,6]. HBCD is stable hydrophobic compounds and persistent in the environment [7]. Toxic effects of HBCD has been described in several publications, and HBCD has been found to disrupt the thyroid system and affect brain development, neuron functions, reproduction, and development [7,8,9]. In May 2013, HBCD was added to the Stockholm Convention on persistent organic pollutants Annex A, and the controls required came into force for most parties to the convention in November 2014 [10]. HBCD has attracted increasing global attentions.

The construction industry is the main user of HBCD, which is incorporated in extruded and expanded polystyrene foam materials. HBCD is also used in textiles in automobile interiors, car cushions, and upholstered furniture and in electric and electronic equipment [11,12]. Commercial HBCD is mainly consist of α-HBCD (10–13%), β-HBCD (1–12%), and γ-HBCD (75–89%) [13]. According to United Nations Environment Program, the annual production of HBCD is approximately 31,000 tons/year. China is the major producer of HBCD in Asia, with an annual output of up to 18,000 tons/year. Of this, 5500–6000 tonnes are exported, 9000 tonnes are used in China in expanded polystyrene foam, and 3000 tonnes are used in China in extruded polystyrene foam [14].

There are a number of pathways through which HBCD can enter the environment, and HBCD has been found in various biological and environmental matrices (including water, sewage sludge, sediment, soil, indoor dust, tree bark, marine mammal tissues, fish, and eggs) around the world [15,16,17,18,19,20]. HBCD is strongly hydrophobic, and sediment has been found to be a sink for HBCD. HBCD concentration and distribution in sediment in China have been investigated in many studies. However, most data on HBCD concentration in sediment in China are for South east and Northeast China, and the concentrations found were less than micrograms per gram of sediment [15,19,21,22,23,24,25,26]. Few studies have been focused on HBCD concentrations in sediment in Northwest China.

The Weihe River is a typical river in Northwest China, which contains arid and semi-arid areas. The Weihe River Basin is the most developed part of Northwest China, and the Weihe River runs through many large cities and industrial areas in Shaanxi Province. China has implemented the “One Belt and One Road” program. Xi’an City, the national cooperation platform of “One Belt and One Road”, locates in the Weihe River basin [27]. Millions of people were living in the Weihe River basin. There are many chemical, electronics manufacturing, and textile enterprises in the Weihe River Basin. Rapid population increases and economic development have led to large quantities of anthropogenic contaminants entering the Weihe River from primary sources in runoff, in industrial and domestic effluent, and through atmospheric deposition. However, information on the level of HBCD in sediment from the Weihe River Basin is very scarce. In this study, we conducted a comprehensive investigation on the levels and profiles of HBCD and its three isomers in surface sediment in the Weihe River basin. Based on the surveying data, ecological risk assessment of HBCD in Weihe River basin was conducted. The obtained data could provide some data support for the global inventory of HBCD, and provide data information for the future work on pollution control and risk assessment.

## 2. Materials and Methods

### 2.1. Chemicals

Native α-HBCD, β-HBCD, and γ-HBCD standard solutions were purchased from AccuStandard (New Haven, CT, USA). ^13^C_12_-labeled α-HBCD, β-HBCD, and γ-HBCD standards were obtained from Wellington Laboratories (Guelph, ON, Canada). Pesticide residue analysis grade *n*-hexane and dichloromethane, were purchased from Honeywell (Morris Plains, NJ, USA). High performance liquid chromatography grade methanol and acetonitrile were purchased from Fisher (Hampton, NH, USA). Silica gel (63–100 μm) was purchased from SunChrom (Friedrichsdorf, Germany).

### 2.2. Sample Collection

**Figure 1:**
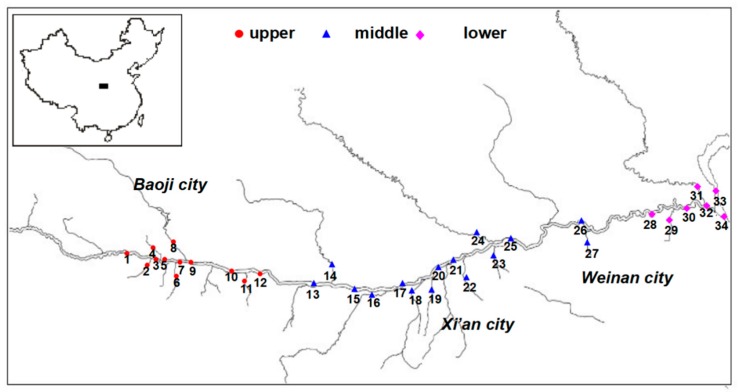
Sampling location of surface sediments in Weihe River basin Shaanxi section, China.

A total of 34 surface sediment samples were collected from the Weihe River Basin in August 2017. The locations of the sampling sites are shown in Figure 1 and Table 1. Of the 34 sampling sites, 15 were on main tributaries of the Weihe River and 19 were on the main Weihe River stream. Sites 1–12 were in the upper reaches of the Weihe River, sites 13–25 were in the middle reaches, and sites 26–34 were in the lower reaches. Each surface sediment sample was taken from the sediment surface to 5 cm underground (0–5 cm) using a stainless steel grab sampler. Each sample was placed in a clean self-sealing aluminum/polyethylene bag with a zip closure. In the laboratory, each sample was dried, ground, and passed through a 60 mesh sieve, then stored in a wide-mouthed bottle.

### 2.3. Sample Extraction and Cleanup

A 20 g aliquot of a sediment sample was spiked with 100 μL (500 ng/mL) of the ^13^C_12_-labeled β-HBCD and γ-HBCD mixture, then Soxhlet extracted with 500 mL mixture of *n*-hexane and dichloromethane (1:1 *v*/*v*) for 16 h. In order to removesulfur, 2.0 g of copper granules was added to extract [21]. The extract was then evaporated to 2.0 mL in a flask using a rotary evaporator (BüchiLabortechnik, Zürich, Switzerland) at 40 °C. The extract was passed through a multi-layer column, from bottom to top as follows: 1.0 g silica gel, 4.0 g alkaline silica gel, 1.0 g silica gel, 8.0 g acidic silica gel, 2.0 g silica gel, and 2.0 g anhydrous Na_2_SO_4_. The column was eluted with a mixture of dichloromethane and hexane (1/1 *v*/*v*). The first 70 mL was discarded, and the second fraction (contained HBCD) was collected for analysis. The cleaned extract was evaporated and transferred into 1.0 mL methanol contained50.0 ng ^13^C_12_-labeled α-HBCD [21,22].

### 2.4. Instrumental Analysis

Theα-HBCD, β-HBCD, and γ-HBCD concentrations were determined by high performance liquid chromatography/triple quadrupole mass spectrometer (HPLC-MS/MS, TSQ Quantum AccessMAX; Thermo Fisher Scientific, Waltham, MA, USA). The HBCD stereoisomers were separated using a BEH C18 column (2.1 × 150 mm i.d., 3.0 μm, Waters, Milford, MA, USA). The column was kept at 40 °C during an analytical run. The injection volume was 10.0 μL. Three mobile phases were used, (A) water, (B) methanol, and (C) acetonitrile, and the flow rate was 0.2 mL/min. The gradient program started with A/B/C25/20/55 (*v*/*v*/*v*) and was ramped to A/B/C10/20/70 *(v*/*v*/*v*) in 12.0 min, then changed to A/B/C0/0/100 (*v*/*v*/*v*) in 0.2 min (maintained for 8 min), finally returned to A/B/C 25/20/55 (*v*/*v*/*v*) and maintained for 9 min.

The triple quadrupole mass spectrometer was operated in selected reaction monitoring (SRM) mode with electrospray negative ionization mode [21,22]. The capillary temperature and capillary spray voltage were 230 °C and 3.0 kV, respectively. The sheath gas was nitrogen, and the pressure was 28 psi, and the auxiliary gas was also nitrogen, and the pressure was 5 psi. The collision gas used to achieve collision-induced dissociation was argon, and the pressure was 1.5 mTorr. The tube lens offsets for HBCD was 70 eV. The [M − H]^−^→ Br^−^ transitions at *m/z* 640.2 → 81.0/642.2 → 81.0 and *m*/*z* 657.2 → 81.0/659.2 → 81.0 were monitored for the native and ^13^C_12_-labeled HBCD stereoisomers, respectively.

### 2.5. Quality Assurance and Quality Control

Isotope-labeled HBCD mixtures (^13^C_12_-labeled α-, β-, and γ-HBCD) were used to calibrate the instrument to allow HBCD to be quantified, using HBCD mixture at the concentrations of 0.01, 0.02, 0.05, 0.1, 0.2, 0.5, and 1 μg/mL to give seven-point standard calibration curves. Seven real samples were analyzed to test the method. Each sample extract was analyzed three times and the mean concentrations calculated. The relative standard deviations were <12%. Solvent blanks and sample blanks that had been subjected to the clean-up procedure were also analyzed. In the blanks, no target compounds were detected. The quantification limit for each analyte was defined as ten times of the signal-to-noise ratio found in the blank. The α-, β-, and γ-HBCD limits of quantification were 0.15, 0.09, and 0.12 ng/g dry weight (dw), respectively. The recovery of the HBCD stereoisomer surrogate standards was 42–136%.

### 2.6. Total Organic Carbon Analysis

A Vario TOC cube system (Elementar, Langenselbold, Germany) was used to measure the total organic carbon (TOC) content of each sediment sample. A 0.2 g aliquot of a sediment sample packed with quartz wool was loaded into the combustion cup. The sample was wetted with 3% phosphoric acid and then heated to 250 °C for 1 min to remove inorganic carbon. Finally, the sample was heated to 900 °C for 9 min in the combustion house and the non-dispersed infrared detection signal was recorded. Each sample extract was analyzed three times, and the relative standard deviations were <5%.

## 3. Results and Discussion

### 3.1. Concentrations and Spatial Distributions

The HBCD stereoisome concentrations in the sediment samples from the Weihe River Basin are shown in Table 1. HBCD were found in 31 (91.2%) of the samples.

The HBCD concentrations ranged from not detected (nd) to 4.04 ng/g dw, and the mean was 0.45 ng/g dw. To the best of our knowledge, this is the first time HBCD have been measured in sediment from Shaanxi Province [28]. These HBCD concentrations were similar to the concentrations that have been found in sediment from Taihu Lake (0.046–2.56 ng/g dw), the Liaohe River (nd–4.02 ng/g dw), and the Shanghai (0.05–6.87 ng/g dw) in China [15,21,24], and from lakes in the UK (0.88–4.80 ng/g dw) [16], the Detroit River and Erie Lake (nd–1.60 ng/g dw) in North America [29], and the Sydney estuary (1.8–5.3 ng/g dw) in Australia [30]. Relatively high HBCD concentrations have been found in sediment from the Dongjiang River and Hunhe River [19,31] in China, Lake Maggiore in Northern Italy [32], and the major rivers in Korea [33] (Table 2). However, the HBCD concentrations found in our samples were somewhat lower than concentrations found in sediment from other parts of China and other parts of the world, such as from the Yangtze River [26], Dagu Dainage Canal, Haihe River and Tianjin Harborat Bohai Bay [25], Japan [34], Norway [35], Spain [36], United Kingdom [37], and the Netherlands [38] (Table 2). This indicates that the Weihe River is relatively lightly contaminated with HBCD although HBCD contamination is of global concern.

**Figure 2:**
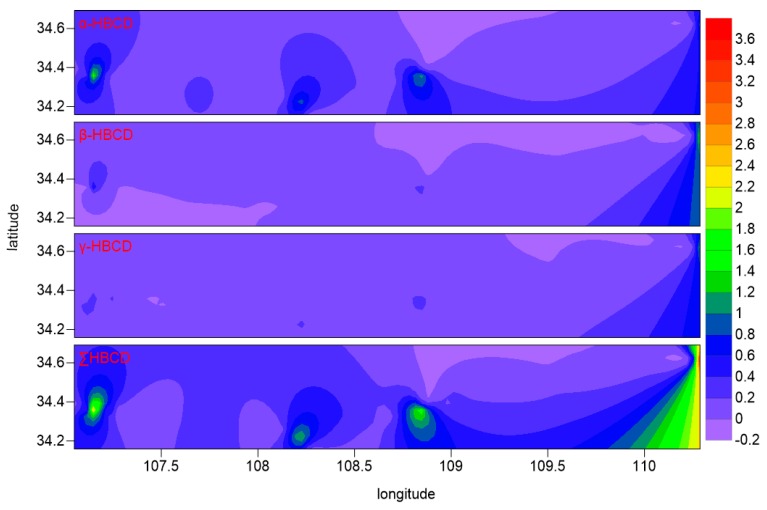
The spatial distribution of HBCD concentration.

The spatial distributions of HBCD concentration in sediment in the study area is shown in Table 1 and Figure 2. Relatively high HBCD concentrations were found in sediment around Baoji City, Xi’an City, and Weinan City. Relatively high HBCD concentration was also found at site S34 in the lower reaches of the Weihe River, possibly because this site is a tourist attraction with many water recreation facilities, which may contain HBCD. Relatively high HBCD concentrations were found at sites S4, S15, and S22, possibly because these sites are close to densely populated and industrial areas. The results demonstrated that local point discharge were important contributors of HBCD in sediment, such as electrical production, waste landfill, sewage discharge, etc.

### 3.2. HBCD Diastereoisomer Compositions

**Figure 3:**
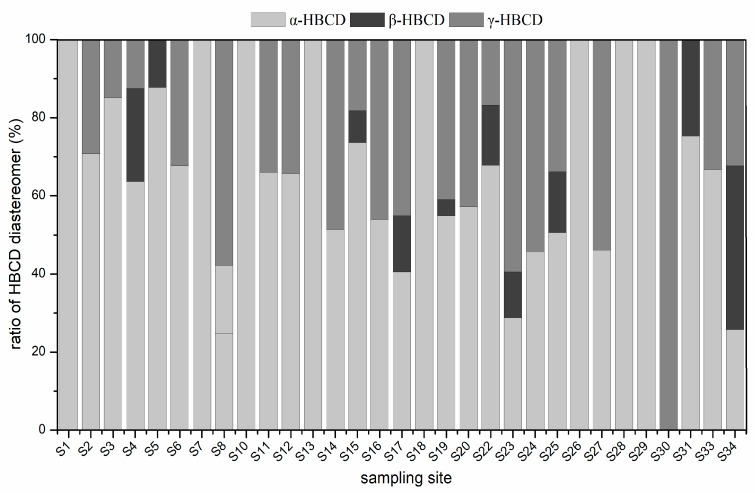
The diastereoisomer profiles of HBCD in sediments of Weihe River.

The α-, β-, and γ-HBCD concentrations in the sediment samples are presented in Table 1. α-HBCD, β-HBCD, and γ-HBCD were detected in 31, 13, and 29 of the all sediment samples, respectively (Table 1). Figure 3 shows the HBCD diastereoisomer profiles in the sediment samples from the Weihe River. As shown in Figure 3, α-HBCD was the dominant diastereoisomer in 22 of the samples, and γ-HBCD and β-HBCD were the dominant diastereoisomers in seven and one of the samples, respectively. The mean contributions of α-, β-, and γ-HBCD to the total HBCD concentrations in the samples were 57.1%, 16.3%, and 38.6%, respectively.

The HBCD diastereoisomer profiles in surface sediment from other regions and countries have generally been similar to the commercial HBCD mixtures (α-HBCD 10–13%, β-HBCD 1–12%, γ-HBCD 75–89%). However, the HBCD diastereoisomer profiles were very different in different samples in our study. Theα-HBCD contributions to the total HBCD concentrations ranged from 24.7% to 87.9%. The α-HBCD contributions in most of the samples were clearly higher than the α-HBCD contributions in commercial HBCD formulations. High α-HBCD contributions have also been found in sediment samples from other locations [22,26,39]. For instance, relatively high α-HBCD contributions of 12.1–100% were found in sediment from three major river drainage basins in Shanghai [24]. β- and γ-HBCD can be bioisomerized intoα-HBCD by organisms, and β- and γ-HBCD have been found more easily metabolized than α-HBCD [40]. α-HBCD is more resistant to biotransformation than β- and γ-HBCD [41]. Meanwhile α-HBCD has a longer half-life [37]. The high α-HBCD contributions to the total HBCD concentrations in our samples may therefore have been caused by the bioisomerization of β- and γ-HBCD into α-HBCD and by α-HBCD being degraded more slowly than γ-HBCD under anaerobic conditions [16,42,43]. Isomer transport, accumulation, and transformation processes in the environment may also have contributed to the high contributions of α-HBCD to the total HBCD concentrations [12,44]. The highα-HBCD contributions may also have been related to α-HBCD released from treated products such as building materials, insulating materials, and textiles in urban environments being washed into rivers. The contributions of γ-HBCD to the total HBCD concentrations in the sediment samples were much lower than the contributions of γ-HBCD to technical HBCD products and slightly lower than the contributions of α-HBCD to the total HBCD concentrations in the sediment samples.

Spearman correlation analyses of the diastereoisomer and total HBCD concentrations were performed, and the results are summarized in Table 3. All the HBCD diastereoisomer concentrations significantly and positively correlated with each other and with the total HBCD concentrations (*p* < 0.01), indicating that the HBCD diastereoisomers had similarsources. The highest correlation coefficient was for the relationship between the β-HBCD and γ-HBCD concentrations.

### 3.3. Correlation Analysis

The organic matter in sediment has a strong adsorption capacity to hydrophobic organic pollutants. TOC content of sediment represents the amount of organic matter. In this study, the TOC contents of the sediment samples were 0.55–3.71%, and the mean was 1.47% (Table 1).

Correlations between the individual HBCD diastereoisomer and total HBCD, and the TOC contents were assessed. As shown in Table 3, a poor but important correlation was found between the α-HBCD concentration and TOC content (r = 0.137), which imply that TOC has a weak effect in the transport and distribution of α-HBCD in sediment. No correlations were found between the concentrations of β-HBCD (r = −0.178), γ-HBCD (r = −0.158), and total HBCD (r = −0.039) and the TOC content, suggesting that sediment TOC content has little influence on the spatial distributions of β-HBCD and γ-HBCD. The sediments from three major rivers in Shanghai and seven major river drainage basins in China also found the similar results [24,26]. This could be explained by the effects of the source proximity, transformation, long-range transport processes, and high contributions of phytoplankton organic carbon to the TOC [45].

### 3.4. Ecological Risk Assessment of HBCD in Sediment

HBCD has been found in natural ecosystems around the world, so the potential effects of these compounds on organisms in sediment, soil, and water have become an important subject for research. Risk quotient (RQ) method was used to perform the ecological risk assessment to estimate the potential toxicity related to exposure HBCD at the concentration that was found in the sediment samples [46,47,48,49]. The RQ method is considered to be the most effective method for assessing the risks posed by contaminants to aquatic organisms. Each RQ was calculated from the predicted no effect concentration (PNEC) and the measured environmental concentration (MEC) using the equation

RQ = MEC/PNEC(1)

PNEC_wat_ = EC_50_/AF (2)

where EC_50_ is the half maximal effective concentration and AF is an assessment factor. The mean measured environmental concentration (HBCD 0.45 ng/g dw) were used as the MECs. The PNEC was calculated from the EC_50_ and the AF. The AF represented possible variations between acute and chronic conditions, and an AF of 1000 was used to represent chronic toxicity in this study [22]. The HBCDEC_50_ values for growth inhibition tests using *Skeletonemacostatum* (72hEC_50_ 52 μg/L) [50] was used.

The risks posed by the pollutants in sediment were assessed using PNECs calculated using Equation (3)

PNEC_sed_ = PNEC_wat_ × K_oc_ × TOC/1000 (3)

where K_oc_ is the sediment–water partition coefficient and TOC is the organic carbon fraction in the sediment. The mean measured TOC value for the sediment samples was 1.47% dry sediment. HBCD K_oc_ values of 45,708.82, were taken from previous publications [51].

Three risk levels were used based on the RQ. RQ > 1 was classed as high risk, 0.1 < RQ < 1 as medium risk, and RQ < 0.1 as low risk. The calculated RQs for HBCD was 0.0129, indicating that HBCD do not pose strong risks to aquatic algae organisms in the Weihe River Basin.

## 4. Conclusions

In our study, the concentration and spatial distribution of HBCD was assessed by determining HBCD in 34 sediments from Weihe River Basin. The levels of HBCD in sediments were relatively lower than other regions worldwide. Relatively high HBCD concentrations were found in the urbanized areas (Baoji, Xi’an, Weinan). The distribution profiles of α-, β-, γ-HBCD were 24.7–87.9%, 0–42.0%, and 0–67.1%, respectively, and α-HBCD dominated in majority sample sites. The high α-HBCD contributions to the total HBCD concentrations maybe caused by the bioisomerization of β- and γ-HBCD intoα-HBCD and by α-HBCD being degraded more slowly than γ-HBCD under anaerobic conditions. The potential risk posed by HBCD in sediment was characterized using the risk quotient method. The achieved RQ < 0.1 and demonstrated that HBCD in sediment in the selected section of the Weihe River present no risk to aquatic algae organisms.

## Figures and Tables

**Table 1:** Information of the sediment samples, total organic carbon (TOC) content and the concentrations of HBCD (ng/g dw).

Sampling Site	Longitude	LATITUDE	TOC (%)	α-HBCD (ng/g dw)	β-HBCD (ng/g dw)	γ-HBCD (ng/g dw)	∑HBCD (ng/g dw)
S1	107°2′59″	34°22′46″	1.33	0.0740	ND	0.0230	0.0970
S2	107°6′26″	34°20′45″	1.78	0.6400	ND	0.2639	0.9039
S3	107°7′39″	34°21′52″	1.54	0.2334	ND	0.0406	0.2740
S4	107°9′15″	34°21′51″	2.16	2.0446	0.7696	0.3972	3.2115
S5	107°11′27″	34°21′11″	1.67	0.3651	0.0508	ND	0.4159
S6	107°15′9″	34°21′6″	2.02	0.0906	ND	0.0433	0.1339
S7	107°15′11″	34°21′6″	0.58	0.0189	ND	0.0130	0.0319
S8	107°18′16″	34°21′56″	1.28	0.1414	0.0997	0.3306	0.5717
S9	107°23′39″	34°20′29″	1.32	ND	ND	ND	ND
S10	107°34′44″	34°17′46″	1.69	0.0785	ND	0.0460	0.1245
S11	107°38′34″	34°16′48″	3.71	0.2716	ND	0.1405	0.4121
S12	107°56′31″	34°14′33″	1.93	0.1114	ND	0.0582	0.1696
S13	108°5′28″	34°14′3″	1.34	0.0593	ND	0.0870	0.1463
S14	108°7′8″	34°15′3″	0.87	0.0293	ND	0.0277	0.0571
S15	108°12′59″	34°12′45″	1.58	1.0083	0.1119	0.2489	1.3691
S16	108°16′21″	34°9′31″	0.67	0.0455	ND	0.0388	0.0843
S17	108°34′4″	34°13′50″	0.79	0.0430	0.0153	0.0479	0.1061
S18	108°34′12″	34°12′17″	2.23	0.0863	0.0160	0.0450	0.1473
S19	108°31′55″	34°17′34″	1.30	0.1957	0.0149	0.1460	0.3566
S20	108°41′53″	34°19′27″	1.56	0.0719	ND	0.0538	0.1257
S21	108°51′25″	34°23′31″	1.03	ND	ND	ND	ND
S22	108°50′42″	34°22′15″	1.21	1.1893	0.2710	0.2939	1.7542
S23	109°0′17″	34°24′16″	1.26	0.0973	0.0400	0.2012	0.3386
S24	108°59′59″	34°23′4″	1.48	0.0371	ND	0.0442	0.0813
S25	109°6″1″	34°28′5″	1.40	0.0680	0.0212	0.0454	0.1346
S26	109°31′32″	34°31′26″	1.49	0.0392	ND	0.0170	0.0562
S27	109°32′4″	34°30′38″	1.77	0.0609	ND	0.0716	0.1325
S28	109°59′46″	34°37′44″	1.37	0.0201	ND	0.0410	0.0611
S29	110°0′17″	34°37′24″	1.68	0.0999	0.0180	0.0390	0.1569
S30	110°7′46″	34°39′48″	1.26	0.0230	ND	0.0418	0.0648
S31	110°8′19″	34°41′28″	1.13	0.0586	0.0192	ND	0.0778
S32	110°11′4″	34°38′23″	1.22	ND	ND	ND	ND
S33	110°15′14″	34°36′43″	1.88	0.0557	ND	0.0277	0.0834
S34	110°17′15″	34°36′43″	0.55	1.0410	1.6986	1.3024	4.0420

ND: not detected.

**Table 2:** Comparison of HBCD concentrations in sediment samples among different regions.

Location	∑HBCD (ng/g dw)	References
Taihu Lake (China)	0.046–2.56	[15]
Shanghai (China)	0.05–6.87	[24]
Liaohe River (China)	nd–4.02	[21]
Dongjiang River (China)	0.03–31.6	[19]
Xijiang River (China)	nd–1.02	[19]
Dayanhe River (China)	0.03–0.61	[19]
Hunhe River (China)	0.05–25.8	[31]
Yangtze River (China)	0.35–206.1	[26]
Haihe River (China)	1.25–26.4	[25]
Dagu Dainage Canal (China)	5.59–634	[25]
Bohai Bay (China)	17.4–244	[25]
English lake (UK)	0.88–4.8	[16]
Erie Lake and Detroit River (USA)	0.26–1.6	[29]
Sydney estuary (Australia)	1.8–5.3	[30]
Maggiore Lake (Italy)	nd–23.7	[32]
Seomjin, Nam, and Nakdong Rivers (Korea)	0.19–13	[33]
Tsurumi (Japan)	5.7–22	[34]
Kuzuryu (Japan)	2.7–20	[34]
Norwegian fjord (Norway)	35–9000	[35]
Cinca River (Spain)	2–42	[36]
Scheldt basin (UK)	0.2–950	[37]
Western Scheldt (UK)	0.6–99	[37]
Scheldt estuary (Netherlands)	14–71	[38]

nd: not detected.

**Table 3:** Results of Pearson correlation analysis between total organic carbon (TOC) and individual HBCD diastereoisomers, and HBCD in sediment samples.

	TOC	α-HBCD	β-HBCD	γ-HBCD	∑HBCD
TOC	1				
α-HBCD	0.137	1			
β-HBCD	−0.187	0.622 **	1		
γ-HBCD	−0.158	0.618 **	0.941 **	1	
∑HBCD	−0.039	0.889 **	0.925 **	0.898 **	1

** Correlation is significant at the 0.01 level.
